# Blue Emitting Star-Shaped and Octasilsesquioxane-Based Polyanions Bearing Boron Clusters. Photophysical and Thermal Properties †

**DOI:** 10.3390/molecules25051210

**Published:** 2020-03-07

**Authors:** Justo Cabrera-González, Mahdi Chaari, Francesc Teixidor, Clara Viñas, Rosario Núñez

**Affiliations:** Institut de Ciència de Materials de Barcelona (ICMAB-CSIC), Campus U.A.B., 08193 Bellaterra, Barcelona, Spain; justocab@ucm.es (J.C.-G.); ch.mahdi39@gmail.com (M.C.); teixidor@icmab.es (F.T.); clara@icmab.es (C.V.)

**Keywords:** boron clusters, boranes, metallacarboranes, triphenylbenzene, octasilsesquioxanes, luminescence

## Abstract

High boron content systems were prepared by the peripheral functionalisation of 1,3,5-triphenylbenzene (TPB) and octavinylsilsesquioxane (OVS) with two different anionic boron clusters: *closo*-dodecaborate (B_12_) and cobaltabisdicarbollide (COSAN). TPB was successfully decorated with three cluster units by an oxonium ring-opening reaction, while OVS was bonded to eight clusters by catalysed metathesis cross-coupling. The resulting compounds were spectroscopically characterised, and their solution-state photophysical properties analysed. For TPB, the presence of COSAN dramatically quenches the fluorescence emission (λ_em_ = 369 nm; Φ_F_ = 0.8%), while B_12_-substituted TPB shows an appreciable emission efficiency (λ_em_ = 394 nm; Φ_F_ = 12.8%). For octasilsesquioxanes, the presence of either COSAN or B_12_ seems to be responsible for ∼80 nm bathochromic shift with respect to the core emission, but both cases show low emission fluorescence (Φ_F_ = 1.4–1.8%). In addition, a remarkable improvement of the thermal stability of OVS was observed after its functionalisation with these boron clusters.

## 1. Introduction

Over the last ten years, a great effort from the scientific community has been done to develop luminescent boron cluster-based materials. The direct or indirect binding of icosahedral boron clusters to luminophores cause a significant effect on the photoluminescent behaviour of the final materials [1,2]. Polyhedral boranes are characterised by their three-dimensional (3D) structure with electrons’ delocalisation inside the cage [3,4]. Among the boron cluster families, the most extensively studied are those of the carborane class. They have a highly polarisable σ-aromatic character and interact electronically with π-conjugated systems [5,6]. The interest in carboranes and metallacarboranes as potential units for molecular materials might be endorsed to their remarkable chemical, thermal, and biological stabilities [7,8,9,10,11,12,13,14,15,16,17]. Owing to the properties above of carboranes, they have been used as exceptional building blocks for developing a large variety of fluorescent carborane-containing molecules, in which the cluster is usually linked to a fluorescent π-conjugated organic system, either directly by the C_c_ [18,19,20,21,22,23,24,25,26,27,28,29,30] or through one spacer [7,8,9,31,32,33,34,35,36,37,38,39].

In addition to the properties described for carboranes, the presence of a cobalt metal centre in the cobaltabisdicarbollide anion (COSAN, [3,3′-Co(C_2_B_9_H_11_)_2_]^-^) confers to these cluster unique redox properties, that can be tuned upon dehydrohalogenation [40,41,42], as well as extraordinary high chemical and thermal stability [43,44,45]. This anion has a high molecular volume, low nucleophilic character, and low charge density because the negative charge is distributed between 45 atoms [46]. The protonated form and sodium salt of COSAN cluster have shown exceptional physicochemical properties such as amphiphilicity, leading to supramolecular interactions and self-assembling in water [47,48,49,50,51,52,53]. All these properties and the biocompatibility of COSAN make this cluster a promising scaffold for biomedical applications [13,54,55,56,57,58,59,60].

From the wide variety of boron clusters, the dodecahydro-*closo*-dodecaborate dianion [61], commonly referred to as *closo*-dodecaborate (*closo*-B_12_, [B_12_H_12_]^2−^), has received increased attention due to its high chemical, thermal, and hydrolytic stability [62,63], as well as good aqueous solubilising agent [44,64]. Moreover, the water solubility of sodium form of *closo*-B_12_ added to its low toxicity [65], makes this anion and its derivatives ideal candidates to design pharmacophores [66,67,68,69]. Additionally, [B_12_H_12_]^2−^ has found applications in material sciences as molecular motors and actuators in nanodevices [70] or energy storage through the design of stable and rechargeable batteries [71]. 

On the other hand, 1,3,5-triphenylbenzene (TPB) is a thermally and photochemically stable fluorophore with π electron-rich properties [72]. With different substitutions at the peripheral phenyl rings, TPB has emerged as one of the most useful building blocks for a wide variety of organic light emitting materials [73]. Previously, our group and others have reported different sets of star-shaped molecules where the TPB acts as the core of carboranyl-functionalised dendrimers that exhibit fluorescence properties [9,74,75]. Moreover, polyanionic Fréchet-type poly(aryl-ether) dendrimers grown from the TPB core were decorated with three, six, or twelve terminal COSAN units; nevertheless contrary to the above carboranyl-containing poly(aryl-ether) dendrimers functionalised with *o*-carborane clusters, these do not show luminescence properties, as a quenching of the fluorescence takes place after functionalisation with COSAN derivatives [76].

Another outstanding family of compounds used as scaffolds are the octasilsesquioxane cubes (T_8_), with general formula [RSiO_1.5_]_8_. Their 3D structure, chemical versatility in functionality, high robustness and thermal stability, as well as their mechanical, electronic or optical properties [77,78,79], make silsesquioxane-based materials very useful in a wide range of applications: Thermally and chemically resistant polymers and ceramics [80,81,82], catalysis [83], nanomedicine [84], or optoelectronic materials [85,86,87], etc. Previously, our group has reported hybrid materials based on octasilsesquioxane structures decorated with terminal *o*-carborane clusters throughout an organic π-conjugated group that exhibit photoluminescence properties (PL) [8]. These systems were synthesised via olefin cross-metathesis reaction [88,89,90], between the octavinylsilsesquioxane (OVS) and several *o*-carboranyl-styrene derivatives bearing different substituents at the adjacent C_c_ atom [8], or by Heck coupling reaction between octa(*p*-bromostyrenyl)silsesquioxane (*p*-BrStyrenylOS) [88] and carboranyl-styrene derivatives [35] in the presence of a palladium catalyst [7]. In general, all these hybrids exhibit moderate to high fluorescent quantum yields in solution; being the most efficient one that contains the nonsubstituted *o*-carborane unit. We concluded that the final PL properties might be tailored by changing the substituents bound to the C_cluster,_ but also the type of carborane isomer. In these hybrids the POSS cage acts as an organising scaffold, causing restriction of the intramolecular movement of the arms, and avoiding additional intramolecular interactions that could cause the fluorescence quenching in the solution. The aforementioned silsesquioxanes-boron cluster hybrids, and another example of carboranyl substituted octasilsesquioxane [91] have shown remarkable thermal stability. We recently reported the first example of octasilsesquioxane cubes bearing COSAN and FESAN clusters [43], however, there are no examples in the literature of octasilsesquioxane structures functionalised with [B_12_H_12_]^2-^. In addition, to the best of our knowledge, the photoluminescence properties of these polyanionic boron rich systems have never been evaluated before.

Herein, we report the synthesis, characterisation, and photophysical properties in solution of two polyanionic star-shaped molecules obtained using 1,3,5-triphenylbenzene (TPB) as a core, which is periphery functionalised with three *closo*-B_12_ or COSAN units. Moreover, a new T_8_-based hybrid decorated with eight *closo*-B_12_ moieties has also been prepared. Furthermore, thermal and photophysical properties of the latter have been compared to its homologous with COSAN (**T_8_-COSAN**), as previously reported [43].

## 2. Results and Discussion

### 2.1. Synthesis and Characterisation

**Scheme 1:**
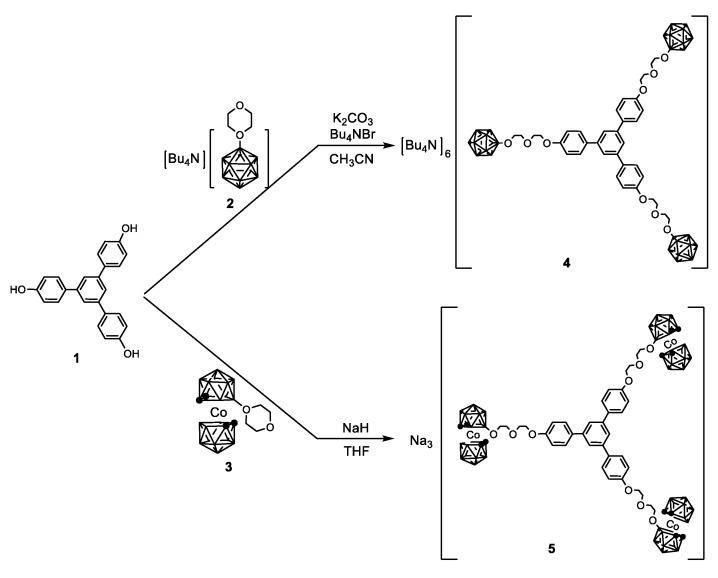
Synthesis of compounds **4** and **5**.

Decoration of the star-shaped core 1,3,5-Tris(4-hydroxyphenyl)benzene (**1**) with the anionic boron clusters was performed via nucleophilic ring-opening reaction of their oxonium-derivatives [92], i.e., [Bu_4_N][B_12_H_11_(C_4_H_8_O_2_)] (**2**) and [3,3′-Co(8-C_4_H_8_O_2_-1,2-C_2_B_9_H_10_)(1′,2′-C_2_B_9_H_11_)] (**3**) [93,94]. Compound **4** was prepared following previously described conditions [95,96,97,98,99,100]: Firstly, deprotonation of hydroxy groups in **1** with K_2_CO_3_ takes place, and secondly, phenoxy moieties undergo a nucleophilic attack promoting the ring-opening reaction of **2**. In a similar manner, compound **5** was synthesised from **1** and COSAN derivative **3** using NaH as a deprotonating agent. In this instance, **5** was obtained as the sodium salt, but Hosmane et al. also have prepared the potassium salt [97]. The reactions were monitored by ^11^B-NMR spectroscopy by comparison with their parent species, and compounds **4** and **5** were isolated in 57% and 63% yield, respectively (Scheme 1). We previously reported the preparation of metallodendrimers based on the same aromatic core **1** functionalised from **3** to 12 COSAN units through hydrosilylation reactions [76], however, the synthetic pathway described here can be considered a simpler and more efficient approach.

**Scheme 2:**
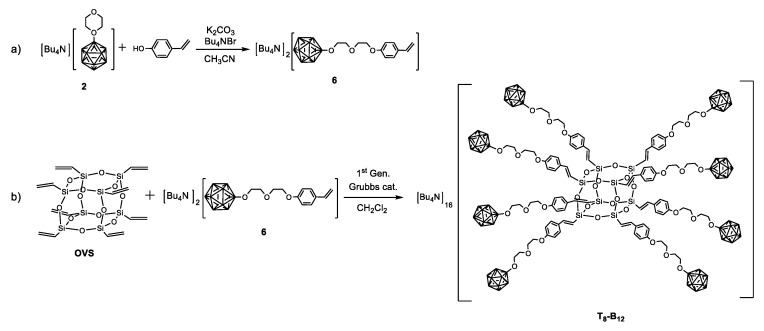
Synthesis of compounds (**a**) **6** and (**b**) **T_8_-B_12_**.

**Figure 1:**
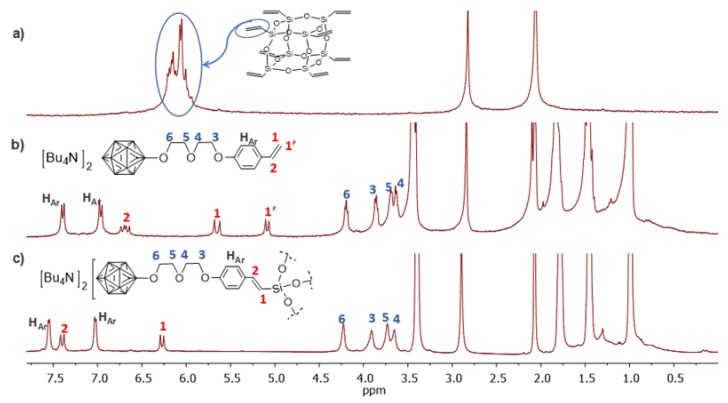
^1^H-NMR spectra in acetone-d_6_ of (**a**) octavinylsilsesquioxane (OVS), (**b**) compound **6** and (**c**) **T_8_-B_12_**.

On the other hand, functionalisation of the octavinylsilsesquioxane (OVS) cube with eight *closo*-dodecaborate units was performed via cross-metathesis reaction, as was previously described for COSAN and FESAN derivatives by our group [43]. Based on that strategy, a new *closo*-dodecaborate precursor bearing a terminal styrene group was prepared by ring-opening reaction of **2** with 4-vinylphenol (Scheme 2a). The evolution of the reaction was also monitored by ^11^B NMR spectroscopy, and compound **6** was obtained in 72% yield. A successful cross-metathesis reaction of OVS and **6** was achieved using the first generation Grubbs catalyst [101] in CH_2_Cl_2_ at reflux for 60 h, and around 20% of the excess of **6** was added to ensure the complete functionalisation of the central cube. The cross-metathesis reaction was monitored by ^1^H NMR upon the disappearance of the vinyl proton resonances from the Si-CH=CH_2_ of OVS (Figure 1). The regio- and stereoselective *E*-isomers of **T_8_-B_12_** was obtained in 18% yield (Scheme 2b). It should be noted that the terminal styrene group in **6** confers to this compound a high chemical versatility to use it as starting material in a variety of reactions.

The molecular structures of all the compounds were established using standard spectroscopy techniques: IR-ATR, NMR (^1^H, ^13^C{^1^H}, and ^11^B{^1^H}) (Figure S2–S17), and elemental analysis.

The IR-ATR spectra for those compounds bearing the *closo*-dodecaborate cluster show the typical υ(B-H) strong bands below 2500 cm^−1^ (**4**, **6,** and **T_8_-B_12_**), while compound **5** exhibit this band around 2534 cm^−1^. For **T_8_-B_12_**, the characteristic broadband around 1090 cm^−1^ due to the vibration frequency of the Si-O bond is also evident (Figure S17). The ^1^H NMR spectra of all compounds show the aromatic resonances corresponding to the phenyl moieties in the region from δ 6.97 to 7.80 ppm, and the -C*H*_2_O- groups are identified in their usual range δ 3.43–4.24 ppm. Additionally, COSAN derivative **5** displays two resonances at 4.29 and 4.32 ppm undoubtedly attributed to the C_c_-*H* protons, while for **6** appears the characteristic vinylic (C*H*=C*H_2_*) distribution as two doublets and one doublet of doublets in the range 5.08–6.69 ppm (Figure 1b). In contrast to **6**, in the ^1^H NMR of **T_8_-B_12_** the vinylic signals have disappeared after the cross-metathesis reaction, giving rise to the new vinylene (C*H*=C*H*) resonances as two doublets with an 18 Hz *trans* coupling constant at 6.26 and 7.39 ppm (Figure 1c). The ^11^B{^1^H} NMR spectrum of **5** shows the typical 1:1:1:1:2:3:3:2:2:1:1 pattern from δ +25 to −28 ppm [95,96,102], and the lowest field resonance is assigned to the [B(8)-O]. For those compounds bearing the *closo*-dodecaborate cluster (**4**, **6,** and **T_8_-B_12_**), the ^11^B{^1^H} NMR spectra show a simpler distribution pattern with a ratio of 1:5:5:1, from +8 to −21 ppm, assigning again the lowest field resonance to the [B(1)-O] [98]. The ^13^C{^1^H} NMR spectra also established the structure of compounds, showing the resonances of aromatic and vinylic carbons for all of them in the region δ 110–160 ppm. Moreover, the terminal vinylic CH=*C*H*_2_* signal at 110.6 ppm displayed for **6** has disappeared after the coupling reaction with OVS, which further confirms the successful synthesis of **T_8_-B_12_**. The ^13^C{^1^H} NMR spectra of the aliphatic ether moieties are shown from δ 67 to 74 ppm, and for compound **5** two additional resonances displayed at 55.2 and 47.2 ppm are attributed to the *C*_c_ from COSAN.

### 2.2. Photophysical Properties

**Figure 2:**
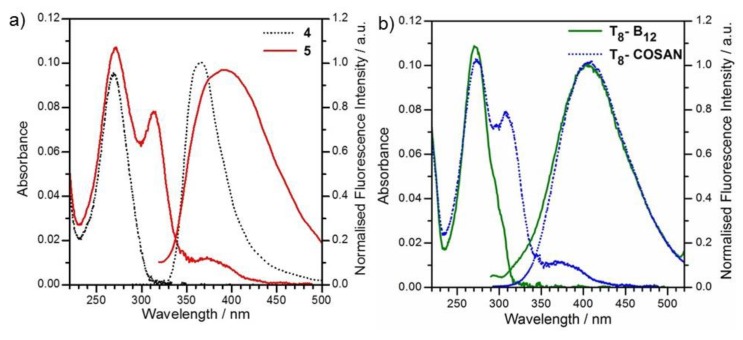
Absorption and emission spectra of (**a**) **4–5** and (**b**) **T_8_-B_12_** and **T_8_-COSAN** recorded in CH_3_CN (au: Arbitrary units).

The solution-state photophysical behaviour of compounds, **4–6**, **T_8_-B_12_**, as well as **T_8_-COSAN** (Figure S1, previously synthesised by us [43]), were studied by UV–Vis and fluorescence (PL) spectroscopy. Results of the optical properties are shown in Figure 2 and listed in Table 1.

Inspecting the absorption spectra of **4** and **5** in CH_3_CN (Figure 2a), high energy bands found around 270 nm are attributed mainly to the π–π* transitions occurring within the aromatic core, which are redshifted compared to the maximum at 254 nm reported for TPB [103,104]. This 270 nm band is the main absorption observed for **4**, but additional bands are identified for COSAN derivative **5**: A band at 313 nm and another near 370 nm, which are endorsed to the metallacarborane unit substituted with the O-(CH_2_)_2_-O-(CH_2_)_2_- moiety [105]. Additionally, a weak band around 450–460 nm due to the d–d transition in Co^III^ is also observed [106]. Molar extinction coefficients (ε) were determined at their higher absorptions, giving a value of 6.8 × 10^4^ M^−1^·cm^−1^ for **4**, which increases up to 8.3 × 10^4^ M^−1^·cm^−1^ for compound **5**, probably due to some contribution of COSAN cluster at that wavelength range [106]. These ε values are also in the range of TPB substituted by hydrosilylation with three COSAN units (ε = 8.6·10^4^ M^−1^·cm^−1^) [76]. Regarding the emission properties, both compounds exhibit a single broadband when excited at their maxima absorption, but a different fluorescence (PL) behaviour is pointed out. In this regard, a remarkable higher PL emission intensity of compound **4**, with a quantum yield value of Φ_F_ = 12.8% is observed, while COSAN derivative **5** do not show appreciable emission, Φ_F_ = 0.8%. The low intensity of emission of compound **5** has been ascribed to a quenching process when the COSAN cluster is chemically bonded to the fluorescent core, which has been previously observed for other COSAN-containing fluorophores [1,76]. Nevertheless, when TPB was substituted with *closo*-carborane units through a -CH_2_- spacer, their Φ_F_ could be modulated from 26% to 55% depending on the substituents at the second C_c_ [9]. In that case, the presence of *closo*-carborane clusters increased the emission efficiency compared to that reported for pristine TPB (Φ_F_ = 10% in CH_2_Cl_2_) [75]. These results confirm that the inherent nature of COSAN induces a notable PL quenching of TPB, while this is not occurring for compound **4** substituted with *closo*-dodecaborate cluster. It should be noted that comparable behaviour was observed when **2** and **3** were appended to organotin fluorophores: While the presence of COSAN reduced the emission efficiency to Φ_F_ ≤ 7%, the substitution of the tin complexes with one *closo*-dodecaborate unit increased this value up to Φ_F_ = 49% [96]. In addition to the different PL emission efficiency of **4** and **5**, the former exhibits a maximum emission (λ_em_) centred at 364 nm, that is in the region of carboranes-bearing TPB [9], but blue-shifted by 25 nm with respect to the weak emission band of **5**.

Similarly, the optical properties of **T_8_-B_12_** and **T_8_-COSAN** were analysed through their UV–Vis absorption and PL spectra in CH_3_CN (Table 1). The UV–Vis spectra of both POSS cages show their absorption maxima as a broadband in the range 270–272 nm, which is endorsed mainly to the styrene moieties [8,43]. Although this band is red-shifted with respect to free styrene (251 nm), this effect is in good agreement with the UV–Vis spectra of previously reported styrenyl-containing POSS with different spacers [8,88]. As described for compound **5**, the presence of COSAN units in **T_8_-COSAN** is responsible for the additional absorption bands near 310 and 370 nm, as well as the weak absorption around 450–460 nm. Regarding the extinction coefficients, the COSAN cluster in **T_8_-COSAN** increases the ε value with respect to **T_8_-B_12_** at their high energy absorption band (from 186,000 to 233,000), due to the nearness of the metallacarborane band at 310 nm. On the other hand, **T_8_-B_12_** and **T_8_-COSAN** show nearly identical PL spectra, with emission maxima at λ_em_ = 405–406 nm after excitation at their maxima absorbance (Figure 2b, Table 1). This emission is in the range to that obtained for carborane-functionalised T_8_ through a styrene group (λ_em_ = 370–414 nm), but it is remarkably redshifted compared to the maximum emission (*λ*_em_ = 326 nm) of (*p*-methoxy-styrenyl)_8_OS [88]. Therefore, the presence of either *closo*-dodecaborate or COSAN clusters as substituents in the styrenylOS cube seems to be responsible for this ∼80 nm bathochromic shift. This could be due to a combination of phenomena, as the high electron delocalisation within the OS cube or possible interactions of the functional groups linked to the cage in the excited state [8,107]. In fact, the emission maxima of **T_8_-B_12_** and **T_8_-COSAN** is even more redshifted than those observed for silsesquioxanes substituted with more conjugated systems, e.g., (stilbene-vinyl)_8_OS emitting in a range λ_em_ = 375–388 nm in different solvents, (*p*-methyl-stilbene-vinyl)_8_OS with λ_em_ = 398 nm [88,108], and even (carborane-vinylstilbene)_8_OS hybrids with λ_em_ = 391–392 nm [7]. Regarding the PL quantum yield, CH_3_CN solutions of **T_8_-B_12_** and **T_8_-COSAN** were analysed. Differently to the equivalent (*p*-methoxy-styrenyl)_8_OS core that shows a Φ_F_ = 12% [88], the polyanionic T_8_ hybrids here prepared show low emission efficiencies, with values Φ_F_ = 1.4–1.8%. It is also worth noting that, in contrast to carborane-substituted T_8_, either through styrene or vinyl-stilbene moieties [8,43], in this work there is no apparent relationship between the type of boron cluster and the emission efficiency behaviour. Nevertheless, it is evident that incorporation of anionic boron clusters to the POSS cube gives rise to low emissive materials in the solution.

### 2.3. Thermal Stability of Anionic-Boron Clusters Containing ***T_8_***

**Figure 3:**
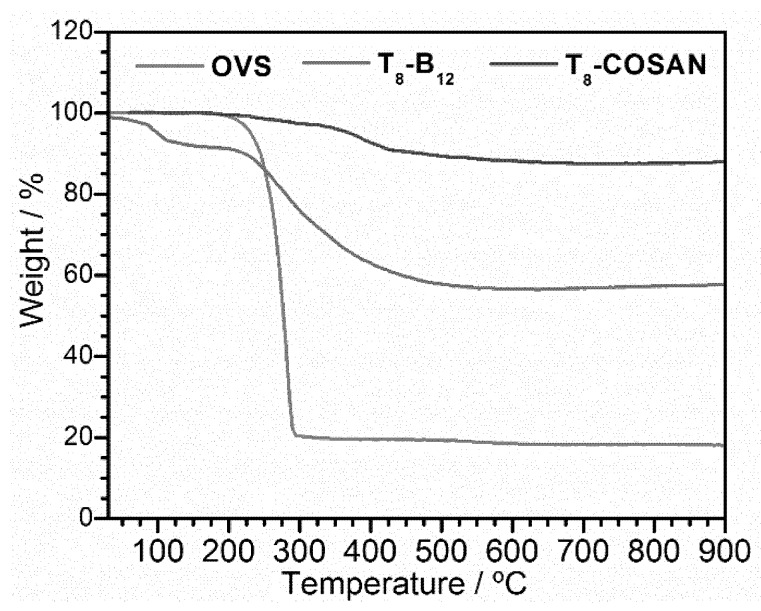
Thermogravimetric analysis (TGA) data under argon (10 °C/min) for octavinylsilsesquioxane (OVS), **T_8_-B_12_** and **T_8_-COSAN**.

Silsesquioxanes have been used as additives in polymers and composites to improve their thermal, mechanical, and oxidative resistance [109,110]. Additionally, it has been stated that boron clusters have high thermal stability (vide supra). To study the influence of thermal behaviour when these two families of compounds are combined, we reported the thermal stability of the **T_8_-COSAN** hybrid [43], and the thermogravimetric analysis (TGA) of **T_8_-B_12_** under argon was here studied (Figure 3). The TGA results show a very different behaviour when nonfunctionalised OVS is compared to **T_8_-B_12_** and **T_8_-COSAN**. Pristine OVS shows a sharp fall near 290 °C, and after heating up to 900 °C, 18% of the initial weight was recovered [111]. On the contrary, the boron cluster-containing OVS hybrids undergo a gradual weight reduction between 300–500 °C, and a residual mass of around 58% and 88% were respectively recovered, after heating up to 900 °C for **T_8_-B_12_** and **T_8_-COSAN**, respectively. In **T_8_-B_12_** the percentage of organic part is around 82%, whereas only 11% would correspond to the percentage of hydrogen. Therefore, after burning these materials, we may propose that the total of hydrogen and a percentage of the organic part (around 31%) are lost; being this percentage higher for **T_8_-B_12_** with regards to **T_8_-COSAN** (around 4.4%). This result confirms once again that binding anionic boron clusters to the T_8_ cube cause a significant increase in the thermal stability of the final materials; moreover, those materials containing metallacarborane fragments are the most thermally stable. 

## 3. Materials and Methods 

### 3.1. Materials

All reactions were performed under an atmosphere of dinitrogen employing standard Schlenk techniques. Acetonitrile and dichloromethane were purchased from Merck and distilled from sodium benzophenone before use. Commercial grade acetone, hexane, ethyl acetate, tetrahydrofuran, and methanol were used without further purification. Compounds [Bu_4_N][B_12_H_11_(C_4_H_8_O_2_)] (**2**) [93] and [3,3′-Co(8-C_4_H_8_O_2_-1,2-C_2_B_9_H_10_)(1′,2′-C_2_B_9_H_11_)] (**3**) [94] and **T_8_-COSAN** [43] were synthesised according to the literature. Compounds [Bu_4_N]Br, Grubbs 1st generation catalyst [RuCl_2_(CHPh)-(PCy_3_)_2_], octavinylsilsesquioxane (**OVS**), 1,3,5-tris(4-hydroxyphenyl)benzene (**1**) and 4-vinylphenol solution 10 wt% in propylene glycol were purchased from Sigma-Aldrich (Merck Life Science, Madrid, Spain). K_2_CO_3_ was obtained from Labkem (Barcelona, Spain). 

### 3.2. Instrumentation

Elemental analyses were performed using a Thermo (Carlo Erba) Flash 2000 Elemental Analyser microanalyzer (EA Consumables, Inc., Pennsauken, NJ, USA). ATR-IR spectra were recorded on a JASCO FT/IR-4700 spectrometer on a high-resolution (Madrid, Spain). The ^1^H-NMR (300.13 MHz), ^11^B{^1^H} (96.29 MHz), and ^13^C{^1^H} NMR (75.47 MHz) spectra were recorded on a Bruker ARX 300 spectrometer (Bellerica, MA, USA). All NMR spectra were recorded in CD_3_COCD_3_ solutions at 25 °C. Chemical shift values for ^11^B{^1^H} NMR spectra were referenced to external BF_3_·OEt_2_, and those for ^1^H and ^13^C{^1^H} NMR were referenced to SiMe_4_ (TMS). Chemical shifts are reported in units of parts per million downfield from the reference, and all coupling constants are reported in Hertz. UV–Vis spectra were recorded on a VARIANT Cary 5 UV–Vis-NIR spectrophotometer (Santa Clara, CA, USA), using a spectroscopic grade ACN (Sigma-Aldrich, Merck Life Science, Madrid, Spain), in normal quartz cuvette having 1 cm path length, for different solutions for each compound in the range 2 × 10^−6^ to 5 × 10^−6^ M in order to calculate the molar extinction coefficients (*ε*). The fluorescence emission spectra and excitation spectra for all samples were recorded in a VARIANT Cary Eclipse fluorescence spectrometer. No fluorescent contaminants were detected on excitation in the wavelength region of experimental interest. The fluorescence quantum yields were determined by the “single point method” and repeated three times with similar optical density for reproducibility [112], against quinine sulfate in a 0.5 M aqueous sulfuric acid with *ϕ*_F_ = 0.54 as a standard [113].

### 3.3. Synthesis of Derivatives ***4, 5, 6,*** and ***T_8_-B_12_***

#### 3.3.1. Synthesis of **4**

A 5 mL round-bottomed flask was charged under nitrogen with **1** (5 mg, 0.014 mmol), **2** (20.90 mg, 0.044 mmol), K_2_CO_3_ (25 mg, 0.181 mmol), and [NBu_4_]Br (15.4 mg, 0.047 mmol) in 2 mL of anhydrous CH_3_CN. The solution was stirred and refluxed overnight. After, the reaction mixture was washed with THF, filtered, and the solvent was removed under vacuum. Further purification was performed by preparative layer chromatography (CH_2_Cl_2_/CH_3_CN, 1:1) to obtain compound **4** as white solid. Yield: 20 mg, 57%. ^1^H NMR, δ (ppm): 7.72 (br, 6H, C_6_*H*_4_), 7.70 (s, 3H, C_6_*H*_3_), 7.08 (d, ^3^*J*(H,H) = 9 Hz, 6H, C_6_*H*_4_), 4.24 (t, ^3^*J*(H,H) = 4.5 Hz, 6H, C*H*_2_-O), 3.94 (t, ^3^*J*(H,H) = 4.5 Hz, 6H, C*H*_2_-O), 3.74 (br, 6H, C*H*_2_-O), 3.69 (br, 6H, C*H*_2_-O), 3.42 (t, ^3^*J*(H,H) = 9 Hz, 48H, N-C*H*_2_), 1.85–1.75 (m, 48H, N-CH_2_-C*H*_2_), 1.51–1.39 (m, 48H, N-CH_2_-CH_2_-C*H*_2_), 0.98 (t, ^3^*J*(H,H) = 7.5 Hz, 72H, N-CH_2_-CH_2_-CH_2_-C*H*_3_); ^11^B{^1^H} NMR, δ(ppm): 7.72 (s, 3B, *B*–O), –15.34 (s, 15B), –16.13 (s, 15B), –20.89 ppm (s, 3B); ^13^C{^1^H} NMR, δ (ppm): 159.94 (s; *C*_6_H_4_), 142.73 (s; *C*_6_H_3_), 134.38 (s, *C*_6_H_4_), 129.16 (s, *C*_6_H_4_), 124.02 (s; *C*_6_H_3_), 116.05 (s; *C*_6_H_4_), 73.96 (s; O–*C*H_2_), 70.31 (s; O–*C*H_2_), 68.93 (s; O–*C*H_2_), 68.66 (s; O–*C*H_2_), 59.59 (s, N*C*H_2_), 24.68 (s, CH_3_CH_2_*C*H_2_), 20.50 (s, CH_3_*C*H_2_CH_2_), 14.06 (s, *C*H_3_); ATR-IR (cm^−1^): ν = 2959, 2931, 2871 (C_ar_H), 2466 (B-H), 1606 (C=C), 1470 (N-C).

#### 3.3.2. Synthesis of **5**

A solution of **1** (68 mg, 0.193 mmol) and NaH (57.7% dispersion, 28 mg, 0.66 mmol) in 5 mL of dry THF was stirred under nitrogen for 1 h at room temperature. Then, **3** (250 mg, 0.61 mmol) was added and the mixture was refluxed for 60 h. The reaction was cooled down and quenched with CH_3_OH (1 mL), water (3 mL), and a few drops of acetic acid (1 M). The organic solvents were removed under vacuum, and the crude product was dissolved in 10 mL diethyl ether and extracted with water (3 × 10 mL). The organic layer was dried over MgSO_4_ and the volatiles were reduced under vacuum. Further purification was performed with preparative layer chromatography (CH_2_Cl_2_/CH_3_CN, 8:2) to obtain **5** as an orange solid. Yield: 201 mg, 63%. ^1^H-NMR, δ (ppm): 7.80 (br, 6H; C_6_*H*_4_), 7.78 (s, 3H, C_6_*H*_3_), 7.11 (d, ^3^*J*(H,H) = 8 Hz, 2H, C_6_*H*_4_), 4.32 (br, 6H, C_c_–*H*), 4.29 (br, 6H, C_c_–*H*), 4.23 (t, ^3^*J*(H,H) = 4 Hz, 6H, OC*H*_2_), 3.88 (t, ^3^*J*(H,H) = 4 Hz, 6H, OC*H*_2_), 3.65–3.62 (m, 12H, OC*H*_2_); ^11^B{^1^H} NMR, δ(ppm): 24.32 (s, 3B, *B*–O), 5.35 (s, 3B, *B*–H), 1.74 (s, 3B, *B*–H), −1.14 (s, 3B, *B*–H), –2.97 (s, 6B, *B*–H), −6.08 (s, 9B, B–H), −6.82 (s, 9B, *B*–H), −15.95 (s, 6B, *B*–H), –19.10 (br, 9B, *B*–H), –27.08 (s, 3B, *B*–H); ^13^C{^1^H} NMR, δ (ppm): 159.68 (s; *C*_6_H_4_), 142.57 (s; *C*_6_H_3_), 134.31 (s, *C*_6_H_4_), 129.01 (s, *C*_6_H_4_), 123.92 (s; *C*_6_H_3_), 115.73 (s; *C*_6_H_4_), 72.80 (s; O–*C*H_2_), 70.19 (s; O–*C*H_2_), 69.20 (s; O–*C*H_2_), 68.39 (s; O–*C*H_2_), 55.22 (s, *C*_c_–H), 47.20 (s, *C*_c_–H); ATR-IR (cm^−1^): *ῦ* = 3039 (C_c_-H), 2924, 2874 (C_ar_H), 2534 (B-H), 1606 (C=C); elemental analysis calcd. (%) for C_48_H_102_B_54_Co_3_Na_3_O_9_: C, 34.88; H, 6.22. Found: C, 34.72; H, 6.53.

#### 3.3.3. Synthesis of **6**

A mixture of **2** (250 mg, 0.531 mmol), K_2_CO_3_ (293 mg, 2.12 mmol), 4-vinylphenol solution (0.62 mL, 0.535 mmol), [NBu_4_]Br (172 mg, 0.531 mmol), and 10 mL of anhydrous acetonitrile under nitrogen was refluxed overnight in a 25 mL round-bottomed flask. The reaction mixture was filtered off and the solvent was removed under vacuum. The brown oil was dissolved in 10 mL of CH_2_Cl_2_ and extracted with water (3 × 10 mL). The organic layer was dried over MgSO_4_ and the volatiles were reduced under vacuum. Further purification was performed by a silica gel column chromatography (ethyl acetate/hexane, 1:1) to obtain compound **6** as a brownish oil. Yield: 317 mg, 72%. ^1^H NMR, δ (ppm): 7.40 (d, ^3^*J*(H,H) = 9 Hz, 2H, C_6_*H*_4_), 6.97 (d, ^3^*J*(H,H) = 9 Hz, 2H, C_6_*H*_4_), 6.69 (dd, ^3^*J*(H,H) = 18 Hz, ^3^*J*(H,H) = 12 Hz, 1H, C*H*=CH_2_), 5.65 (d, ^3^*J*(H,H) = 18 Hz, 1H, CH=C*H*_2_), 5.08 (d, ^3^*J*(H,H) = 12 Hz, 1H, CH=C*H*_2_), 4.19 (t, ^3^*J*(H,H) = 4.5 Hz, 2H, C*H*_2_-O), 3.86 (t, ^3^*J*(H,H) = 4.5 Hz, 2H, C*H*_2_-O), 3.69 (br, 2H, C*H*_2_-O), 3.62 (br, 2H, C*H*_2_-O), 3.43 (t, ^3^*J*(H,H) = 7.5 Hz, 16H, N-C*H*_2_), 1.87–1.76 (m, 16H, N-CH_2_-C*H*_2_), 1.52–1.40 (m, 16H, N-CH_2_-CH_2_-C*H*_2_), 0.99 (t, ^3^*J*(H,H) = 7.5 Hz, 24H, N-CH_2_-CH_2_-CH_2_-C*H*_3_); ^11^B{^1^H} NMR, δ(ppm): 7.43 (s, 1B, *B*–O), –15.51 (s, 5B), −16.10 (s, 5B), −21.08 ppm (s, 1B); ^13^C{^1^H} NMR, δ (ppm): 159.15 (s, *C*-O), 136.49 (s, *C*H-C_6_H_4_), 130.18 (s, CH-*C*_6_H_4_), 127.33 (s, *C*_6_H_4_), 114.66 (s, *C*_6_H_4_), 110.61 (s, CH=*C*H_2_), 73.13 (s, *C*H_2_-O), 69.22 (s, *C*H_2_-O), 67.90 (s, *C*H_2_-O), 67.90 (s, *C*H_2_-O), 58.63 (s, N-*C*H_2_), 23.74 (s, N-CH_2_-*C*H_2_), 19.51 (s, N-CH_2_-CH_2_-*C*H_2_), 13.10 (s, N-CH_2_-CH_2_-CH_2_-*C*H_3_); ATR-IR (cm^−1^): ν = 2462 (s, B-H st), 1600 (m, C=C st); elemental analysis calcd. (%) for C_44_H_98_B_12_N_2_O_3_ (+3.5 H_2_O + 0.5 CH_2_Cl_2_ + 1 AcOEt): C, 56.74; H, 11.19; N, 2.73. Found: C, 56.70; H, 11.09; N, 2.69.

#### 3.3.4. Synthesis of **T_8_-B_12_**

A 10 mL round-bottomed flask was charged under nitrogen with **OVS** (15 mg, 0.024 mmol), compound **6** (190 mg, 0.227 mmol), and the first generation Grubbs catalyst (12 mg, 0.014 mmol) in 6 mL of CH_2_Cl_2_. The solution was stirred and refluxed for three days. The solvent was removed under vacuum. The residue was treated with a mixture of THF/MeOH (1:10) to obtain a grey solid. Further purification was performed by preparative layer chromatography (acetone/hexane, 7:3) to obtain compound **T_8_-B_12_** as a white solid. Yield: 32 mg, 18%. ^1^H NMR, δ (ppm): 7.54 (d, ^3^*J* (H,H) = 6 Hz, 16H, C_6_*H*_4_), 7.39 (d, ^3^*J* (H,H) = 18 Hz, 8H, C*H*=CH-Si), 7.02 (d, ^3^*J*(H,H) = 9 Hz, 6H, C_6_*H*_4_), 6.26 (d, ^3^*J*(H,H) = 18 Hz, 8H, CH=*C*H-Si), 4.23 (br, 16H, C*H*_2_-O), 3.91 (br, 16H, C*H*_2_-O), 3.72 (br, 16H, C*H*_2_-O), 3.66 (br, 16H, C*H*_2_-O), 3.38 (t, ^3^*J*(H,H) = 9 Hz, 128H, N-C*H*_2_), 1.82–1.72 (m, 128H, N-CH_2_-C*H*_2_), 1.50–1.38 (m, 128H, N-CH_2_-CH_2_-C*H*_2_), 0.98 (t, ^3^*J*(H,H) = 6 Hz, 192H, N-CH_2_-CH_2_-CH_2_-C*H*_3_); ^11^B{^1^H} NMR, δ(ppm): 7.76 (br, 8B, *B*–O), −15.29 (s, 40B), −16.15 (s, 40B), −20.91 ppm (br, 8B); ^13^C{^1^H} NMR, δ (ppm): 160.20 (s, *C*-O), 148.93 (s, *C*H-C_6_H_4_), 129.87 (s, CH-*C*_6_H_4_), 128.46 (s, *C*_6_H_4_), 114.89 (s, *C*_6_H_4_), 114.00 (s, Si-*C*H=CH), 73.10 (s, *C*H_2_-O), 69.32 (s, *C*H_2_-O), 68.08 (s, *C*H_2_-O), 67.92 (s, *C*H_2_-O), 58.53 (s, N-*C*H_2_), 23.71 (s, N-CH_2_-*C*H_2_), 19.53 (s, N-CH_2_-CH_2_-*C*H_2_), 13.20 (s, N-CH_2_-CH_2_-CH_2_-*C*H_3_); ATR-IR (cm^−1^): ν = 2959, 2932, 2872 (C_ar_H), 2466 (B-H), 1602 (C=C), 1479 (N-C), 1091 (Si-O).

## 4. Conclusions

A set of 1,3,5-triphenylbenzene and octasilsesquioxane-based hybrids decorated with three (**4**, **5**) and eight *closo*-decahydro-dodecaborate and cobaltabisdicarbollide (**T_8_-B_12_**_,_ **T_8_-COSAN**), respectively, have been successfully synthesised, isolated, and fully characterised. Although they possess different types of fluorophores, all of them show a similar maxima absorption wavelength, which is red-shifted with regard to the nonsubstituted scaffolds. The molar extinction coefficient is correlated with the type of boron cluster, and proportional to the number of clusters attached to the core molecules. It is worth noting that a significant red-shift of the emission maxima (λ_em_ 369–406 nm) up to 80 nm for the T_8_ hybrids, as well as an important drop of the fluorescence efficiencies were produced after linking these anionic boron clusters to both scaffolds. These results confirm once again that the B_12_ and COSAN clusters produce a significant quenching of the fluorescence in the solution. Notably, binding anionic boron clusters to the OVS provide materials with an extraordinary thermal stability.

## Figures and Tables

**Table 1:** Solution-state photophysical data.

Compounds	*λ*_abs/_nm	*ε*/M^−1^ cm^−1^	*λ*_em_ (*λ*_exc_)/nm	*ϕ*_F_^a^	Stokes Shift (nm)
**4**	269	68000	369 (269)	0.128	100
**5**	272	83000	394 (272)	0.008	122
**T_8_-B_12_**	270	186000	405 (270)	0.014	136
**T_8_-COSAN**	272	233000	406 (272)	0.018	138

^a^ Reference compound quinine sulfate (0.5 M H_2_SO_4_, *ϕ*_F_ = 0.54).

## Supplementary Materials

The following are available online, Figure S1: Structure of compound **T_8_-COSAN**; Figure S2: ^1^H NMR (acetone-d_6_, 300 MHz) of **4**; Figure S3: ^11^B{^1^H} NMR (acetone-d_6_, 300 MHz) of **4**; Figure S4: ^13^C{^1^H} NMR (acetone-d_6_, 300 MHz) of **4**; Figure S5: ^1^H NMR (acetone-d_6_, 300 MHz) of **5**; Figure S6: ^11^B{^1^H} NMR (acetone-d_6_, 300 MHz) of **5**; Figure S7: ^13^C{^1^H} MR (acetone-d_6_, 300 MHz) of **5**; Figure S8: ^1^H NMR (acetone-d_6_, 300 MHz) of **6**; Figure S9: ^11^B{^1^H} NMR (acetone-d_6_, 300 MHz) of **6**; Figure S10: ^13^C{^1^H} NMR (acetone-d_6_, 300 MHz) of **6**; Figure S11: ^1^H NMR (acetone-d_6_, 300 MHz) of **T8-B12**; Figure S12: ^11^B{^1^H} NMR (acetone-d_6_, 300 MHz) of **T8-B12**; Figure S13: ^13^C{^1^H} NMR (acetone-d_6_, 300 MHz) of **T8-B12**; Figure S14: FTIR-ATR spectrum of **4**; Figure S15: FTIR-ATR spectrum of **5**; Figure S16: FTIR-ATR spectrum of **6**; Figure S17: FTIR-ATR spectrum of **T_8_-B_12_**.
