# Supplementary material for: Blue Emitting Star-Shaped and Octasilsesquioxane-Based Polyanions Bearing Boron Clusters. Photophysical and Thermal Properties
Source: Molecules. 2020 Mar 7;25(5):1210. doi: 10.3390/molecules25051210 (PMC7179457; doi:10.3390/molecules25051210)
Supplement: Supplementary file 1 [file molecules-25-01210-s001.pdf]

Blue emitting star-shaped and octasilsesquioxane-based polyanions bearing boron clusters. Photophysical and thermal properties.†

Justo Cabrera-González, Mahdi Chaari, Francesc Teixidor, Clara Viñas and Rosario Núñez*

Institut de Ciència de Materials de Barcelona (ICMAB-CSIC), Campus U.A.B., 08193, Bellaterra, Barcelona, Spain.

* Correspondence: rosario@icmab.es; Tel.: +34-93-580-1853

Contents:

**Figure S1.**	Structure of compound **T_8-COSAN**.
**Figure S2.**	^{1}H NMR (Acetone-d_6, 300 MHz) of **4**.
**Figure S3.**	^{11}B{^{1}H} NMR (Acetone-d_6, 300 MHz) of **4**.
**Figure S4.**	^{13}C{^{1}H} NMR (Acetone-d_6, 300 MHz) of **4**.
**Figure S5.**	^{1}H NMR (Acetone-d_6, 300 MHz) of **5**.
**Figure S6.**	^{11}B{^{1}H} NMR (Acetone-d_6, 300 MHz) of **5**.
**Figure S7.**	^{13}C{^{1}H} NMR (Acetone-d_6, 300 MHz) of **5**.
**Figure S8.**	^{1}H NMR (Acetone-d_6, 300 MHz) of **6**.
**Figure S9.**	^{11}B{^{1}H} NMR (Acetone-d_6, 300 MHz) of **6**.
**Figure S10.**	^{13}C{^{1}H} NMR (Acetone-d_6, 300 MHz) of **6**.
**Figure S11.**	^{1}H NMR (Acetone-d_6, 300 MHz) of **T_8-B_{12}**.
**Figure S12.**	^{11}B{^{1}H} NMR (Acetone-d_6, 300 MHz) of **T_8-B_{12}**.
**Figure S13.**	^{13}C{^{1}H} NMR (Acetone-d_6, 300 MHz) of **T_8-B_{12}**.
**Figure S14.**	FTIR-ATR spectrum of **4**.
**Figure S15.**	FTIR-ATR spectrum of **5**.
**Figure S16.**	FTIR-ATR spectrum of **6**.
**Figure S17.**	FTIR-ATR spectrum of **T_8-B_{12}**.

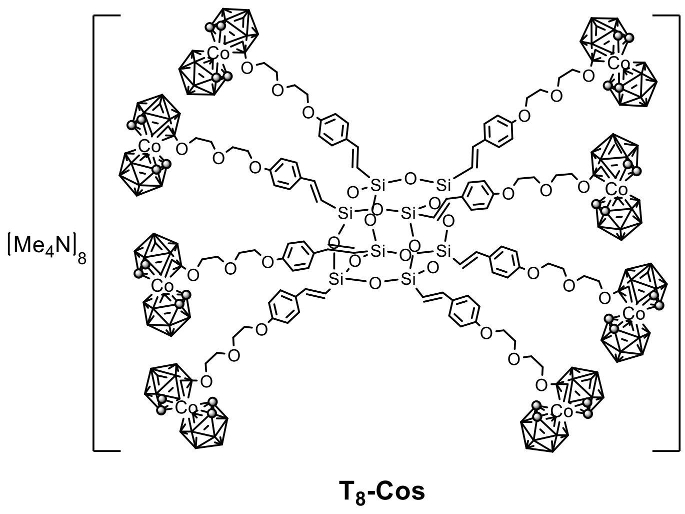

Figure S1. Structure of compound **T_8-COSAN**.

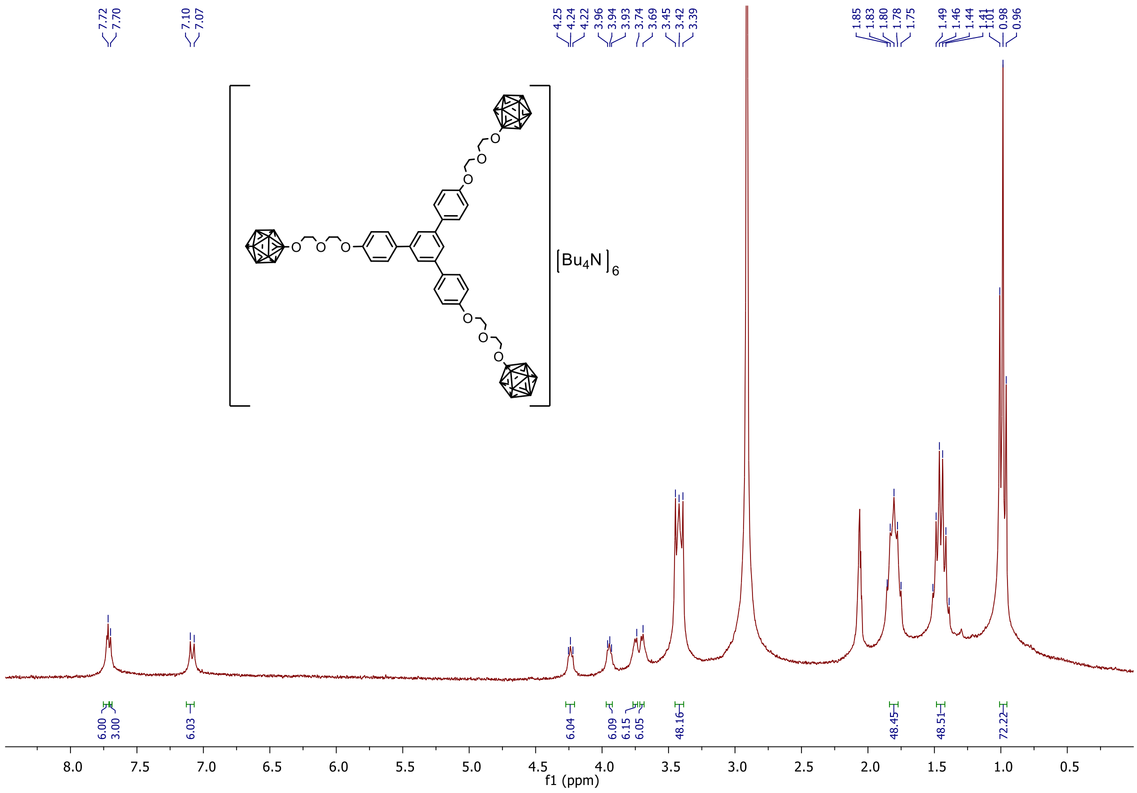

Figure S2. ^{1}H NMR (Acetone-d_6, 300 MHz) of **4**.

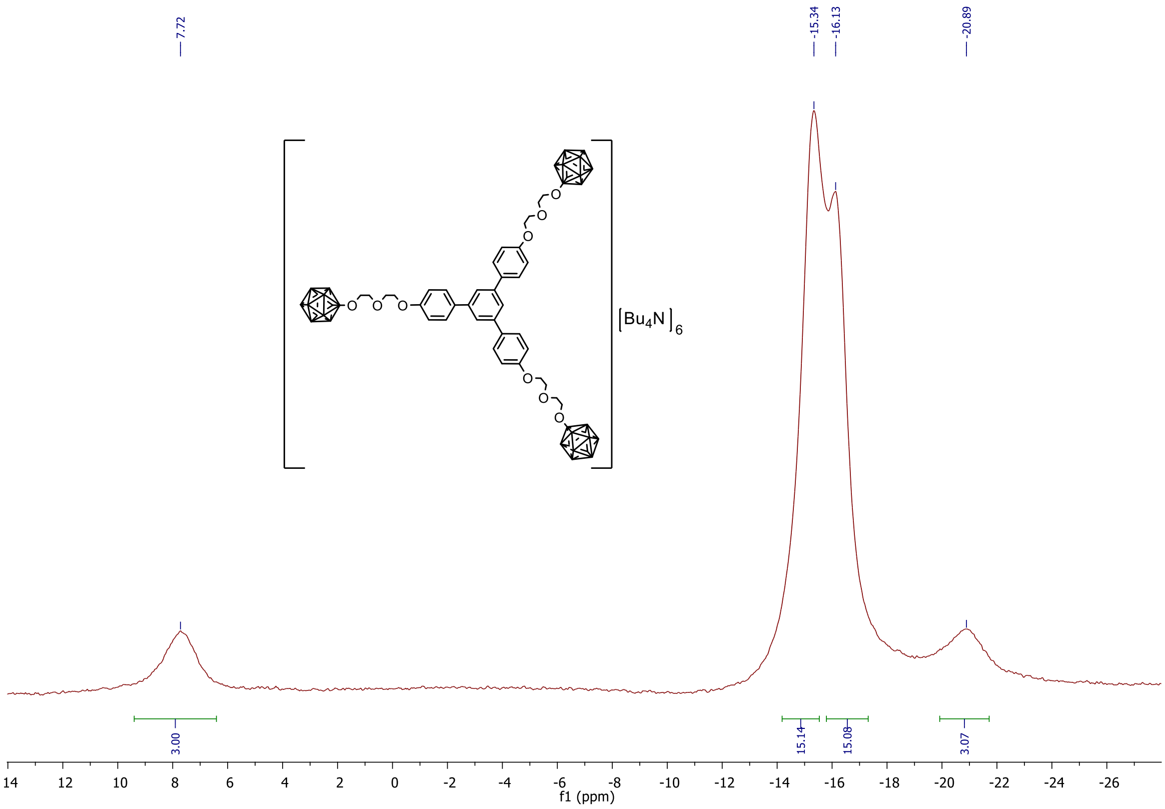

Figure S3. $^{11}B\{^1H\}$ NMR (Acetone-d$_6$, 300 MHz) of **4**.

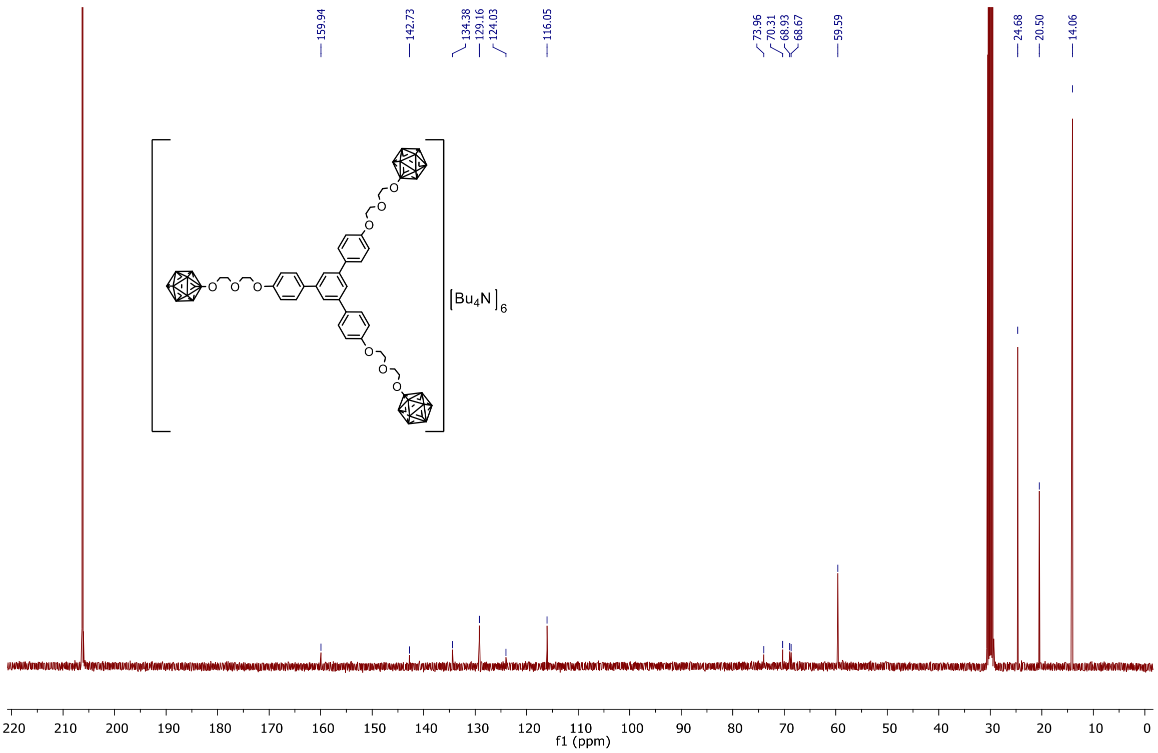

Figure S4. $^{13}C\{^{1}H\}$ NMR (Acetone-d$_6$, 300 MHz) of **4**.

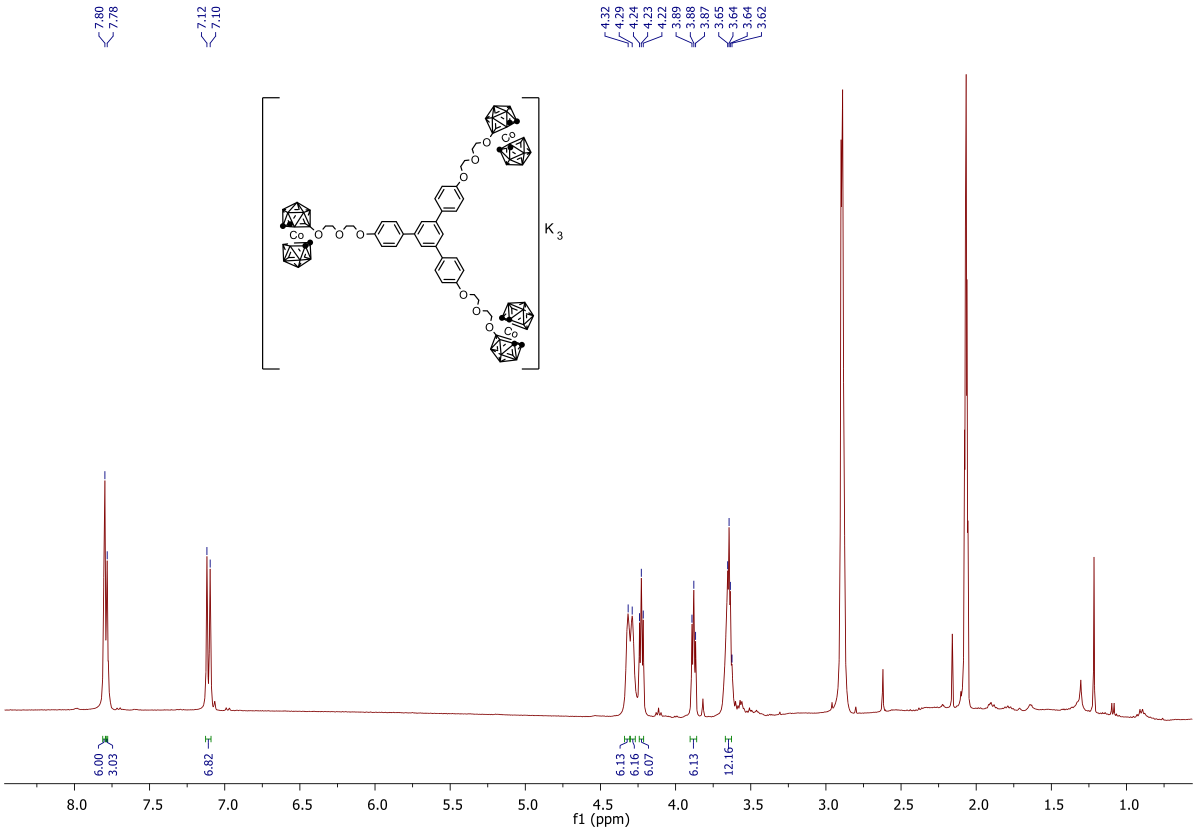

Figure S5. ^{1}H NMR (Acetone-d$_6$, 300 MHz) of **5**.

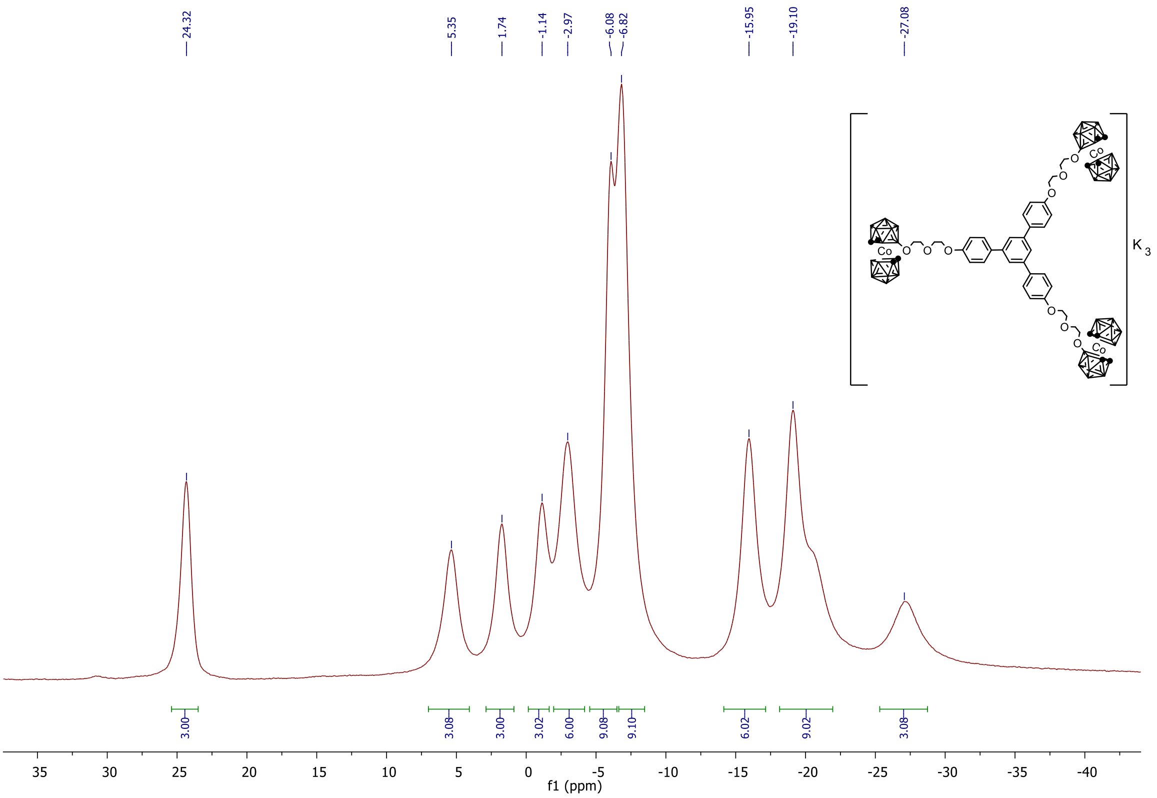

Figure S6. $^{11}B\{^{1}H\}$ NMR (Acetone-d$_6$, 300 MHz) of **5**.

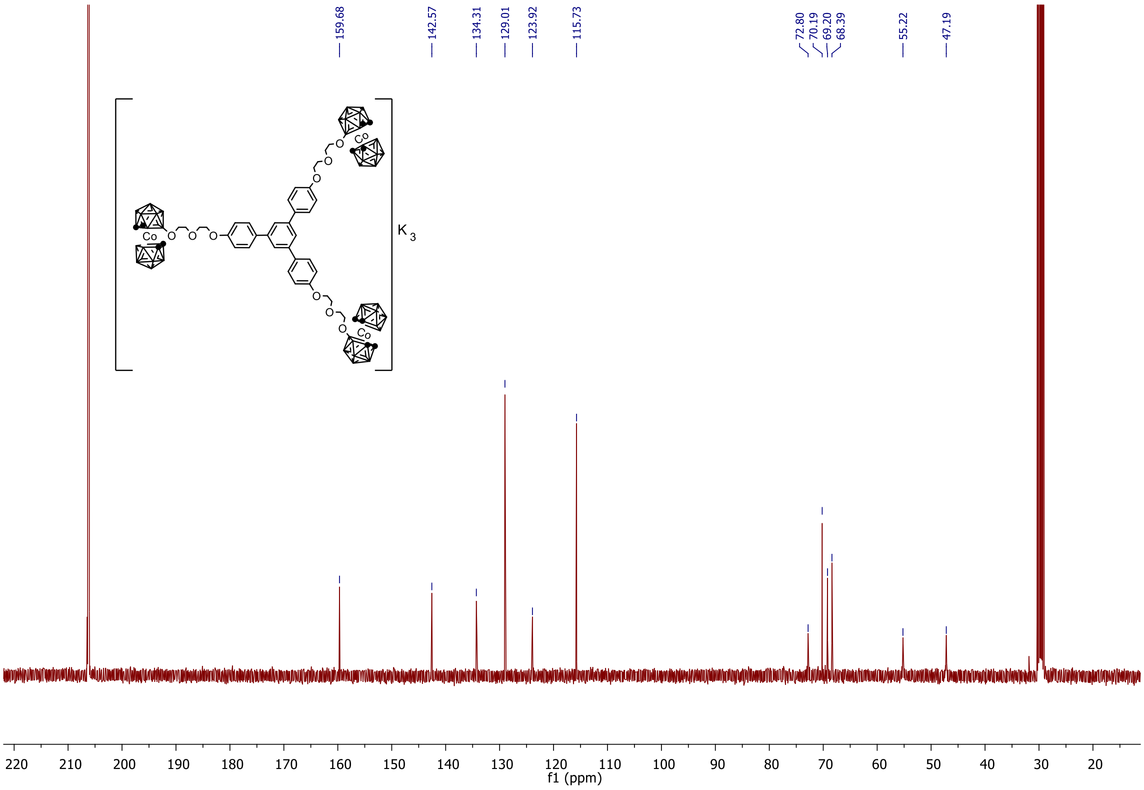

Figure S7. $^{13}C\{^{1}H\}$ NMR (Acetone-d$_6$, 300 MHz) of **5**.

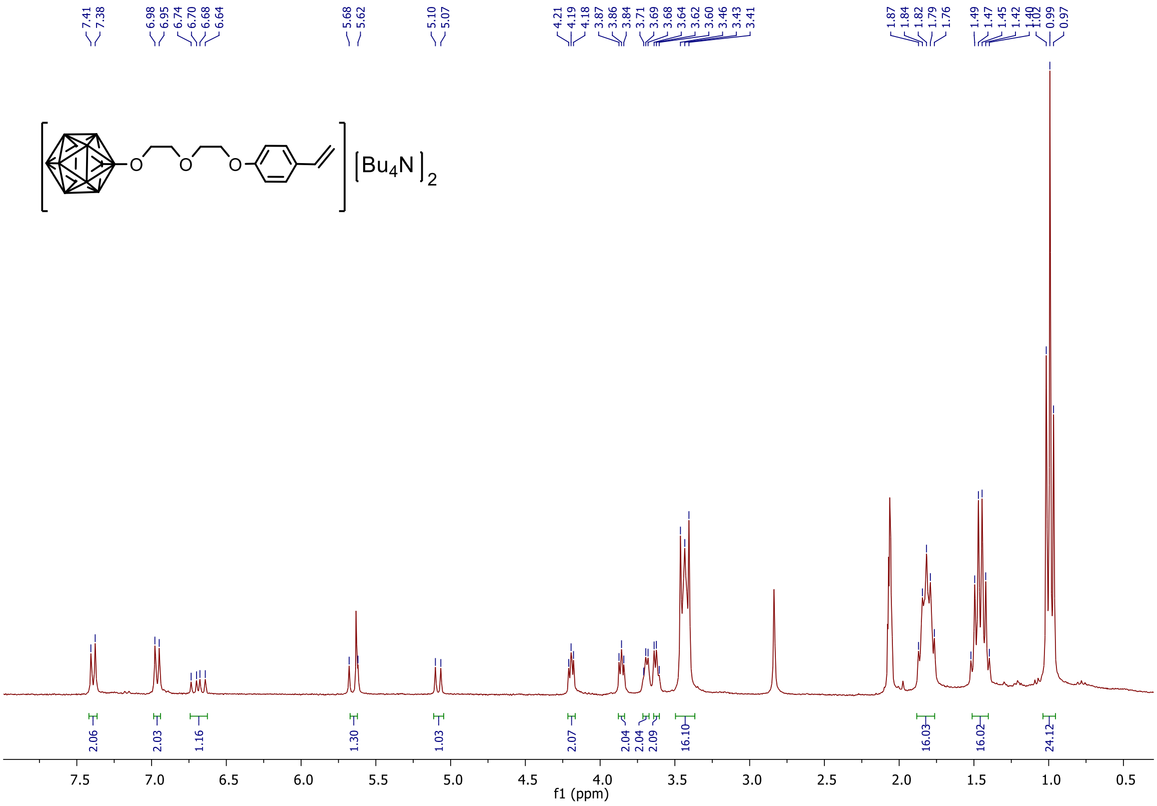

Figure S8. ^{1}H NMR (Acetone-d$_6$, 300 MHz) of **6**.

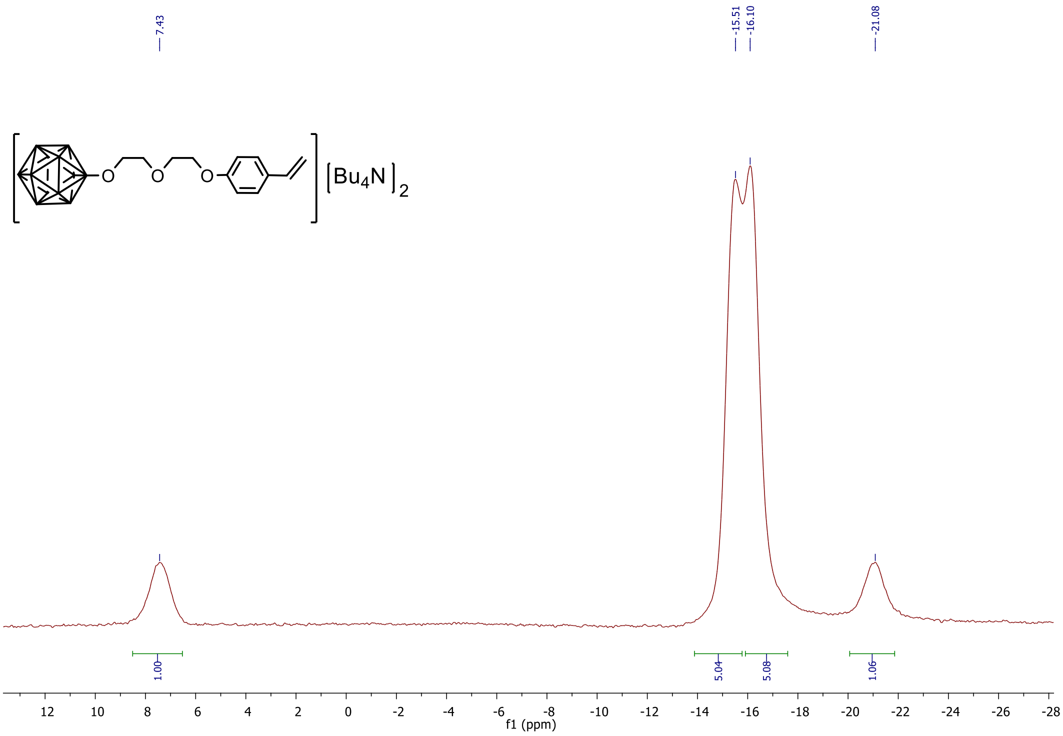

Figure S9. $^{11}B\{^1H\}$ NMR (Acetone-d$_6$, 300 MHz) of **6**.

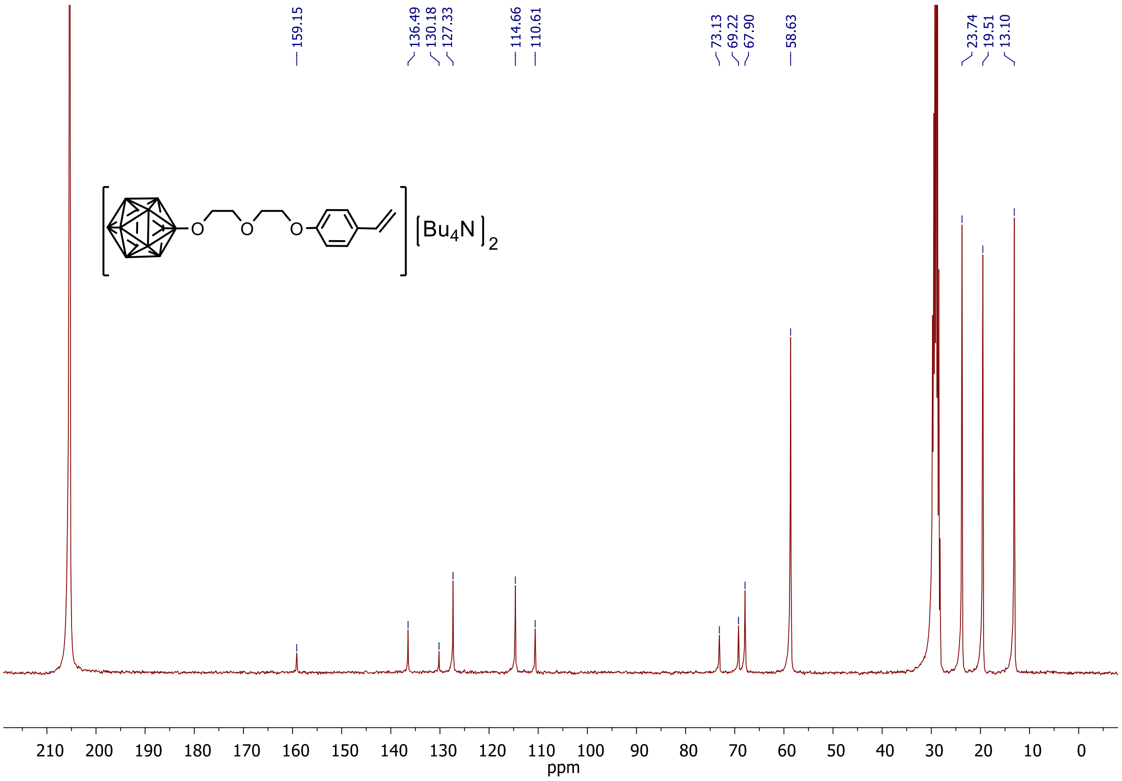

Figure S10. $^{13}C\{^{1}H\}$ NMR (Acetone-d_6, 300 MHz) of **6**.

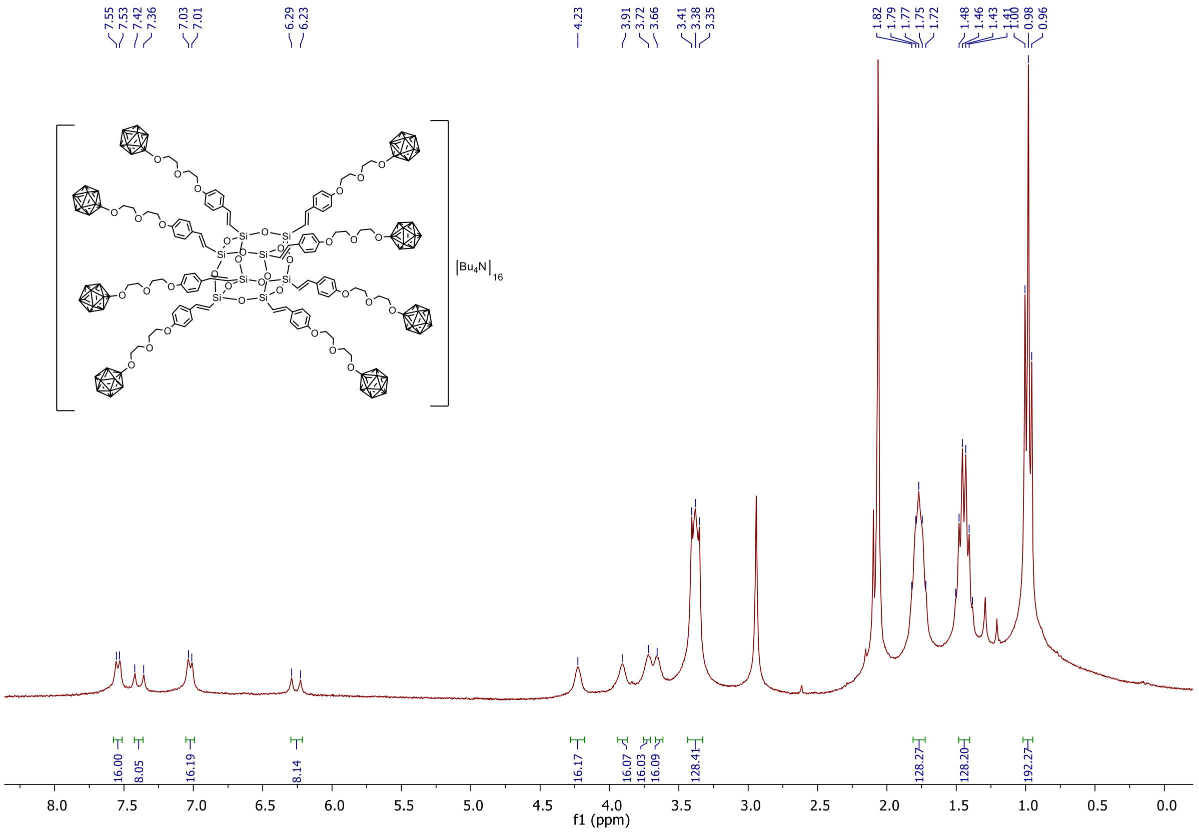

Figure S11. ^{1}H NMR (Acetone-d$_6$, 300 MHz) of **T_8-B_{12}**.

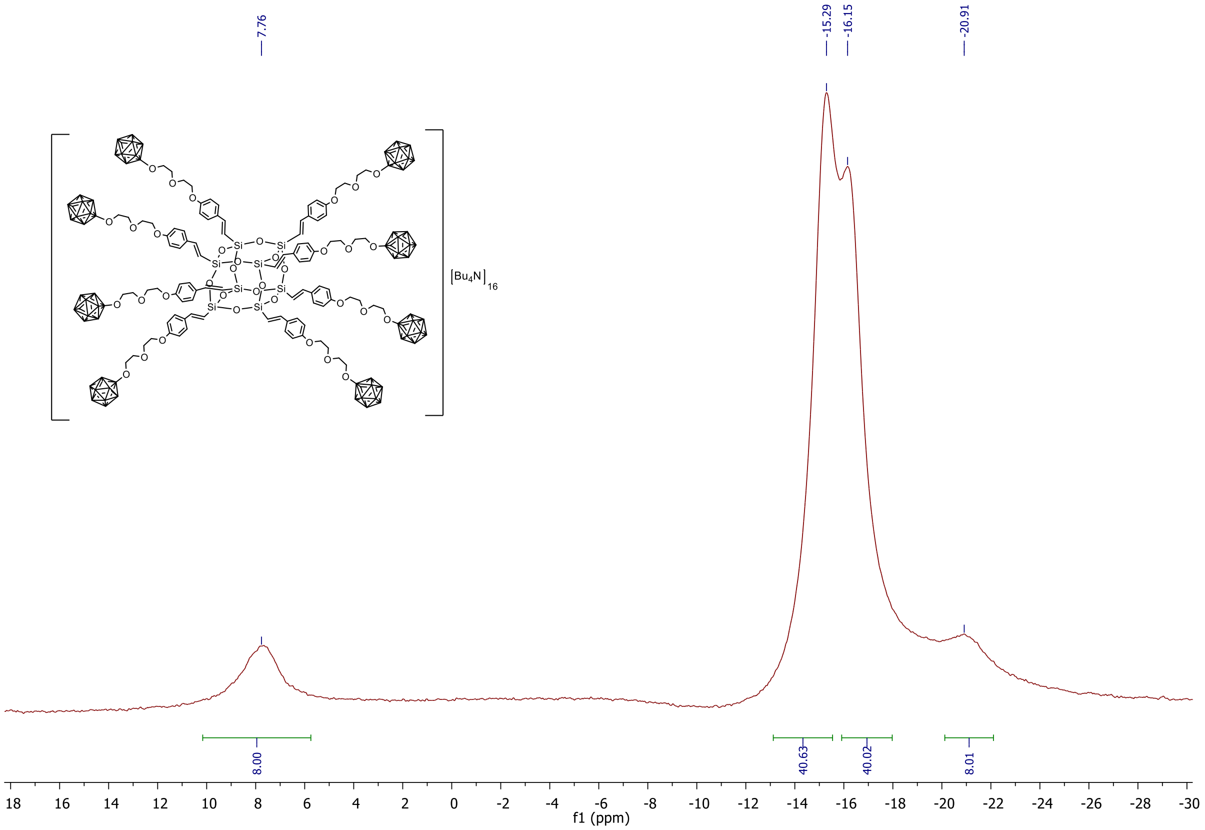

Figure S12. $^{11}B\{^{1}H\}$ NMR (Acetone-d_6, 300 MHz) of **T_8-B_{12}**.

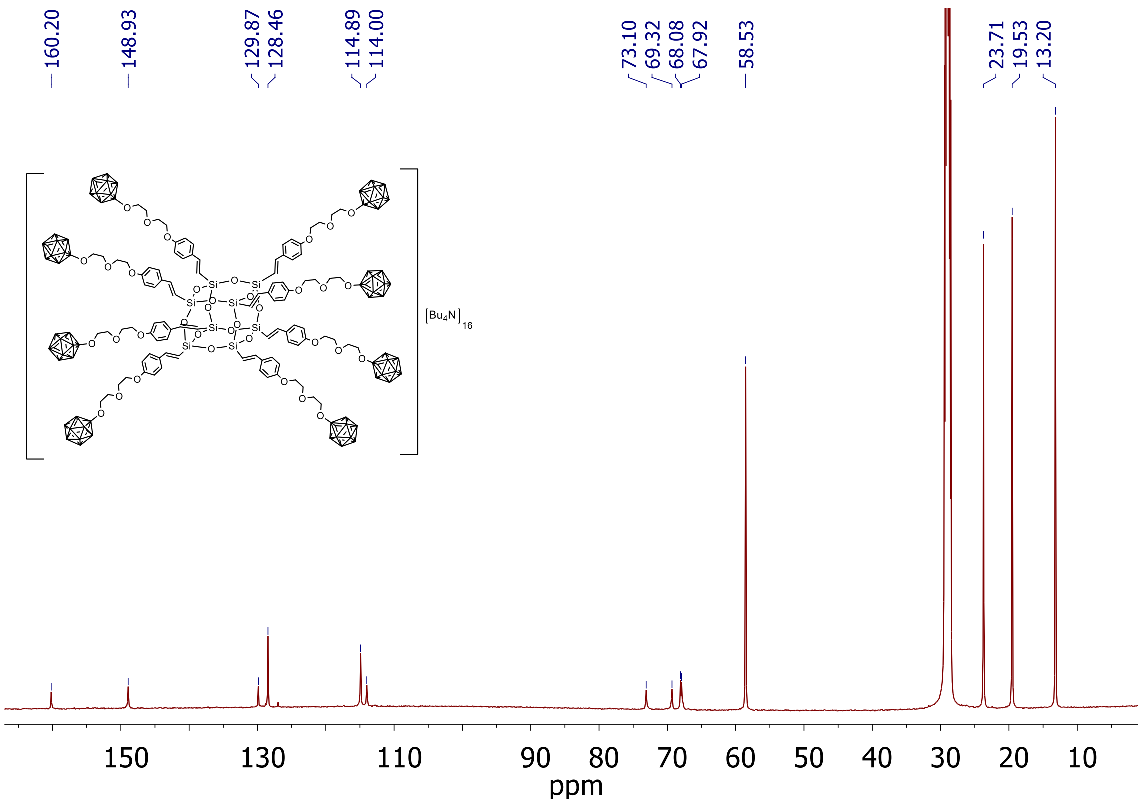

Figure S13. $^{13}C\{^1H\}$ NMR (Acetone-d$_6$, 300 MHz) of **T$_8$-B$_{12}$**.

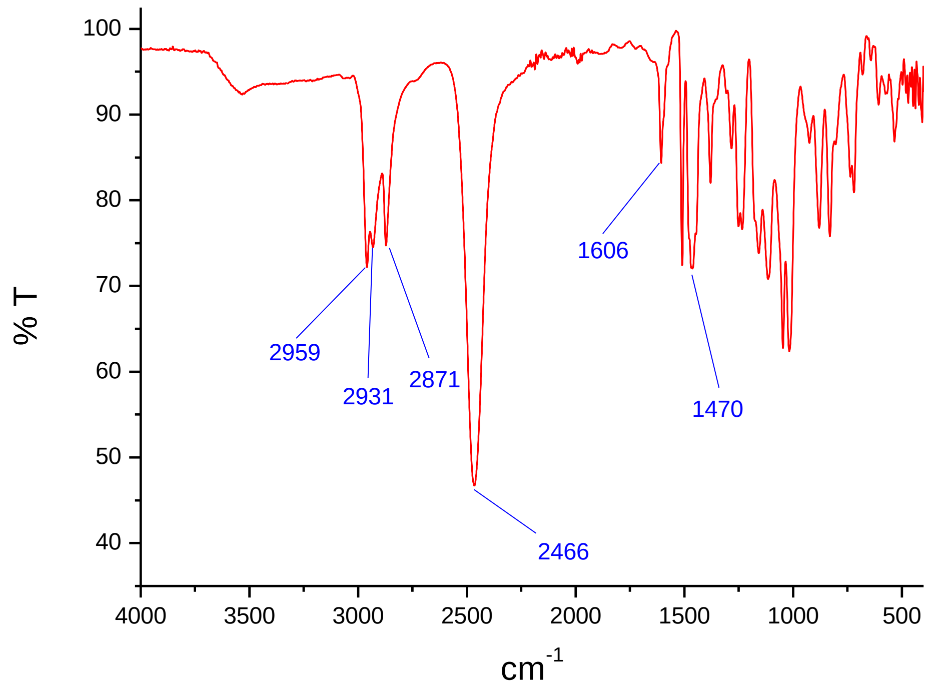

Figure S14. FTIR-ATR spectrum of **4**.

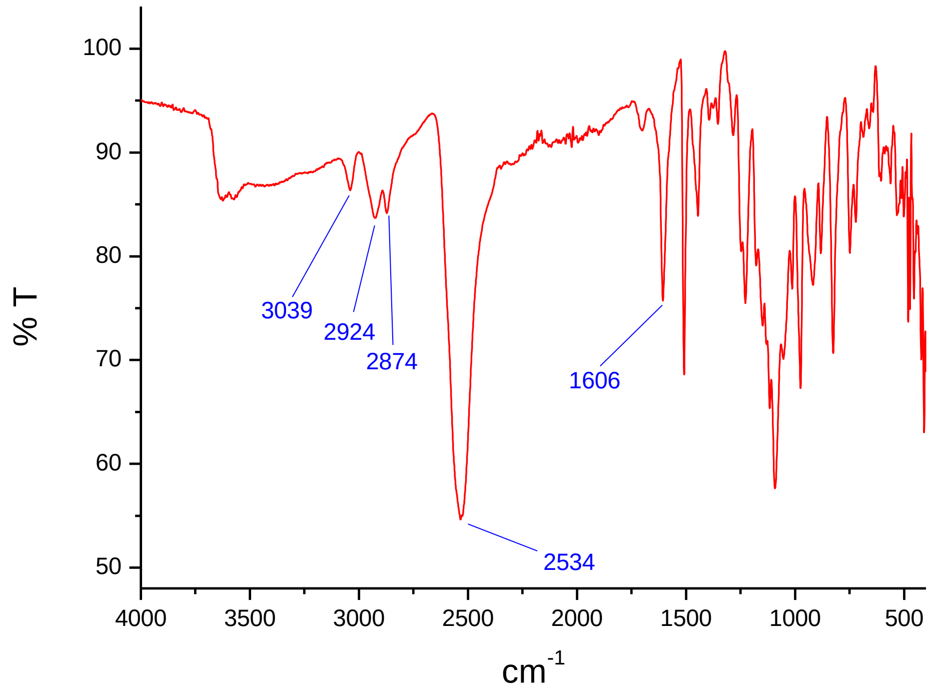

Figure S15. FTIR-ATR spectrum of **5**.

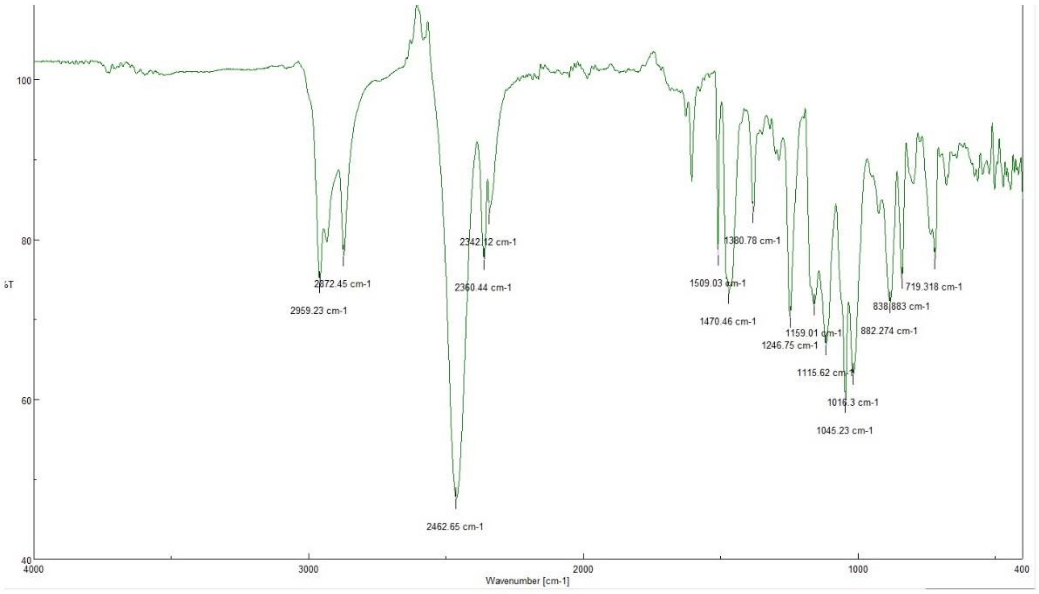

Figure S16. FTIR-ATR spectrum of **6**.

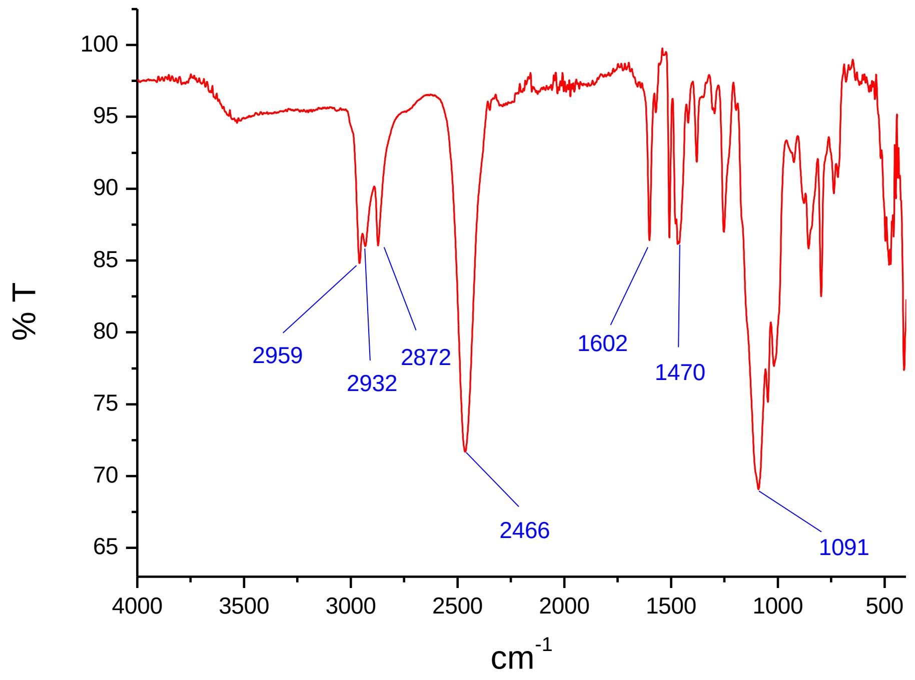

Figure S17. FTIR-ATR spectrum of $\mathbf{T_8}$-$\mathbf{B_{12}}$.
